# Relationship between sleep efficacy endpoints and measures of functional status and health‐related quality of life in participants with narcolepsy or obstructive sleep apnea treated for excessive daytime sleepiness

**DOI:** 10.1111/jsr.13210

**Published:** 2020-10-13

**Authors:** Terri E. Weaver, Susan D. Mathias, Ross D. Crosby, Morgan Bron, Shay Bujanover, Diane Menno, Kathleen F. Villa, Christopher Drake

**Affiliations:** ^1^ Department of Biobehavioral Nursing Science Center for Sleep and Health Research College of Nursing; Division of Pulmonary, Critical Care, Sleep & Allergy, Department of Medicine, College of Medicine University of Illinois at Chicago Chicago IL USA; ^2^ Division of Pulmonary, Critical Care, Sleep & Allergy Department of Medicine College of Medicine University of Illinois at Chicago Chicago IL USA; ^3^ Health Outcomes Solutions Winter Park FL USA; ^4^ Sanford Center for Bio‐Behavioral Research Fargo ND USA; ^5^ Jazz Pharmaceuticals Palo Alto CA USA; ^6^ Jazz Pharmaceuticals Philadelphia PA USA; ^7^ Henry Ford Health System Detroit MI USA

**Keywords:** correlates, functional impairment, hypersomnolence disorders, JZP‐110, sleep‐disordered breathing

## Abstract

This study examined the correlation between improvements in excessive daytime sleepiness in participants with obstructive sleep apnea or narcolepsy and changes in functional status, work productivity and health‐related quality of life. Data from two 12‐week randomized controlled trials of solriamfetol were analyzed. Participants completed the Epworth Sleepiness Scale, 10‐item Functional Outcomes of Sleep Questionnaire, Work Productivity and Activity Impairment questionnaire and 36‐Item Short Form Health Survey and performed the Maintenance of Wakefulness Test at baseline and weeks 4, 8 and 12. Patient Global Impression of Change was assessed at weeks 4, 8 and 12. Pearson correlations were calculated for change in scores from baseline to week 12. For both studies, changes in the 10‐item Functional Outcomes of Sleep Questionnaire were highly correlated (absolute value >0.5) with changes in Epworth Sleepiness Scale scores; changes in multiple domain scores of the 36‐Item Short Form Health Survey and Work Productivity and Activity Impairment questionnaire were moderately correlated (0.3–0.5) with changes in Epworth Sleepiness Scale scores in both studies and highly correlated for participants with narcolepsy. Changes in Maintenance of Wakefulness Test scores correlated moderately with changes in Epworth Sleepiness Scale scores in both studies. At week 12, Patient Global Impression of Change ratings correlated highly with Epworth Sleepiness Scale and 10‐item Functional Outcomes of Sleep Questionnaire scores for both disorders. Other correlations were low. Self‐reported assessments of sleepiness and global improvement appear to be more strongly correlated with measures of functioning and health‐related quality of life than objectively assessed sleepiness.

## 1 INTRODUCTION

Individuals with obstructive sleep apnea (OSA) and narcolepsy often experience excessive daytime sleepiness (EDS) (Fernandez‐Mendoza et al., 2015; Krahn et al., 2015), and as a result may experience fatigue, a decline in work performance, difficulty staying awake while driving, and impairment in focus and personal relationships (Al‐Lawati, 2018; D’Ambrosio et al., 1999; Mulgrew et al., 2007; National Sleep Foundation, 2020; Ulfberg et al., 1996; Ward et al., 2013). EDS can also worsen functionality and social engagement in older adults (Gooneratne et al., 2003; Lee et al., 2013). The standard of care for OSA is primary airway therapy with continuous positive airway pressure (CPAP), although treatment may include oral appliances, hypoglossal nerve stimulation, behavioral treatment options (e.g., weight loss) and surgery (Epstein et al., 2009; Strollo et al., 2014). For many patients, primary airway therapy is not sufficient to resolve EDS; it has been estimated that 9%–22% of those treated with CPAP have persistent EDS (Gasa et al., 2013; Pepin et al., 2009; Weaver et al., 2007). Pharmacotherapy can be used as an adjunct to treatment with CPAP to address EDS (Avellar et al., 2016). There are a variety of pharmacological treatments for EDS in narcolepsy (Billiard et al., 2006; Keam & Walker, 2007; Ruoff et al., 2016), including stimulants, dopamine reuptake inhibitors and dopamine‐norepinephrine reuptake inhibitors.

Measuring the efficacy of pharmacological and non‐pharmacological treatments for EDS in patients with OSA and narcolepsy typically involves evaluating changes in measures of sleepiness or the ability to stay awake. However, there are limited data on how improvements in measures of sleepiness impact functioning, productivity and health‐related quality of life (HRQoL), and how closely, if at all, these distinct concepts are correlated with one another. These analyses sought to examine whether improvements in daytime sleepiness (as measured by the Epworth Sleepiness Scale [ESS] and the Maintenance of Wakefulness Test [MWT]) are associated with changes in functional status, work productivity and HRQoL as assessed by several patient‐reported outcome measures. Analyses also explored the relationship between Patient Global Impression of Change (PGI‐C) ratings and measures of EDS, functioning, work productivity and HRQoL.

## 2 METHODS

### 2.1 Study population and data sources

Two separate studies were conducted with patients with EDS; one included patients whose EDS was associated with OSA, whereas the other involved those with narcolepsy. Each study ensured that enrolled participants’ EDS was due to that study’s qualifying condition (i.e., for those in the narcolepsy trial, their EDS could not be due to OSA). Each study was a phase 3, multicenter, randomized, 12‐week, double‐blind study (Schweitzer et al., 2019; Thorpy et al., 2019) of solriamfetol, a dopamine and norepinephrine reuptake inhibitor approved in the United States and European Union to improve wakefulness in adults with EDS associated with OSA or narcolepsy (Sunosi, 2019, 2020). Both were trials of adults (aged 18–75 years) diagnosed with narcolepsy (type 1 or type 2) or OSA with a baseline ESS score ≥10 and body mass index from 18 to <45 kg/m^2^. Eligible participants also were required to have baseline mean sleep latency <25 (narcolepsy) or <30 (OSA) min on the MWT and usual nightly total sleep time ≥6 h. Key exclusion criteria included usual bedtime later than 1:00 a.m., occupation requiring nighttime or variable shift work, or any other clinically relevant medical, behavioral or psychiatric disorder associated with EDS. For the OSA study, participants had been diagnosed according to International Classification of Sleep Disorders‐3 criteria and reported current or prior use of a primary airway therapy, including a CPAP machine, oral appliance or surgical intervention (Schweitzer et al., 2019). Key exclusion criteria included usual bedtime later than 1:00 a.m., occupation requiring nighttime or variable shift work, or any other clinically relevant untreated medical, behavioral or psychiatric disorder (Schweitzer et al., 2019; Thorpy et al., 2019). Both studies were approved by institutional review boards or ethics committees at each site and performed in accordance with the Declaration of Helsinki; all participants provided written informed consent.

In both studies, participants completed several patient‐reported outcome measures at baseline and again at weeks 4, 8 and 12, including the ESS and multiple measures of functioning and HRQoL. The ESS is a self‐administered questionnaire to measure the level of daytime sleepiness (Johns, 1991). The MWT assesses the ability to stay awake for a defined period of time while seated in a comfortable chair in a darkened room (Mitler et al., 1982). Participants also completed the PGI‐C at each post‐baseline assessment, which is a single‐item rating that assesses the change in the participant’s condition from the start of treatment using a 7‐point scale ranging from 1 (very much improved) to 7 (very much worse). Because the PGI‐C measures change, it was not completed at baseline but was completed at every post‐baseline visit. The 10‐item version of the Functional Outcomes of Sleep Questionnaire (FOSQ‐10) is an abbreviated version of the FOSQ (Weaver et al., 1997) and assesses the impact of EDS on the ability to carry out activities of daily living (Chasens et al., 2009). Like the FOSQ, the FOSQ‐10 is scored to provide a Total score and five subscale scores: General Productivity, Activity Level, Vigilance, Social Outcomes and Intimacy. The Work Productivity and Activity Impairment (WPAI) questionnaire (Reilly et al., 1993) is a six‐item questionnaire that produces four types of scores: the percentage of work time missed due to a problem (Absenteeism); the percentage of impairment experienced while working due to a problem (Presenteeism); the percentage of overall work impairment that is due to a problem (Work Impairment), which represents an aggregate of Absenteeism and Presenteeism; and the percentage of activity impairment that is due to a problem (Activity Impairment), which measures impairment in activities outside of work, during the past 7 days. The WPAI can be adapted to a specific health condition by replacing the word ‘problem’ throughout with the name of the condition; ‘OSA’ and ‘narcolepsy’ were used as the specific condition in each respective study. Work‐related questions (i.e., measures of absenteeism, presenteeism and work impairment) are evaluated in participants who are currently employed, whereas activity impairment is evaluated in all participants. The 5‐Level EuroQoL 5‐Dimension (EQ‐5D‐5L) (EuroQol Research Foundation, 2019) is a five‐item generic measure assessing health status regarding mobility, self‐care, performance in usual activities, pain/discomfort and anxiety/depression. Responses to these items produce a single index value for health status (Utility Index value); there is also a single‐item visual analog scale in which respondents are asked to rate their current health status on a scale from ‘the best health you can imagine’ to ‘the worst health you can imagine’. The Medical Outcomes Study 36‐Item Short Form Health Survey version 2 (SF‐36v2) (Hays & Stewart, 1992; Ware & Sherbourne, 1992) is a 36‐item generic measure assessing vitality, physical functioning, bodily pain, general health, role physical, role emotional, social functioning and mental health. Two summary scores are also calculated (Physical Component Summary and Mental Component Summary scores).

### 2.2 Statistical analysis

Data from the two studies (NCT02348593 and NCT02348606) were analyzed separately, both without regard to treatment assignment. The analyses were based upon the modified intent‐to‐treat population that includes data from participants who were randomly assigned, received at least one dose of study medication, and had a baseline and at least one post‐baseline assessment.

Pearson correlation coefficients were calculated, pooling across all treatment groups, between the ESS, MWT and the FOSQ‐10, EQ‐5D, SF‐36 and WPAI scores separately at baseline, the end of treatment, and as changes from baseline to the end of treatment. Correlations were also calculated between these measures and PGI‐C at the end of treatment. Some measures are scored so that a higher score represents better functioning or lesser severity (FOSQ‐10, EQ‐5D, SF‐36v2, MWT, PGI‐C), whereas other measures are scored so that a higher score represents worse functioning or greater severity (ESS, WPAI). As a result, some correlations are negative and others are positive. When interpreting their magnitude, correlations with absolute values of 0.10 to <0.30, 0.30 to 0.50, and >0.50 are reported as low, moderate and high correlations, respectively (Cohen, 1988). Correlation coefficients were not adjusted for multiple comparisons; therefore, *p*‐values presented are nominal.

## 3 RESULTS

### 3.1 Participant characteristics

The analyses included a total of 690 participants across the two studies (Table 1). The OSA study involved 459 participants, 63% of whom were male, 76% white and 97% from North America, with a mean age of 54 years. Participants with OSA had a mean (standard deviation [SD]) ESS score of 15.2 (3.32) and a mean (SD) FOSQ‐10 score of 13.9 (3.01). The narcolepsy study involved 231 participants, 65% of whom were female, 80% white and 81% from North America, with a mean age of 36 years. Participants with narcolepsy had a mean (SD) ESS score of 17.2 (3.18) and a mean (SD) FOSQ‐10 score of 11.7 (3.03). At the baseline evaluation, 64% of participants with OSA and 61% of participants with narcolepsy were currently employed and provided responses to ≥ 1 of the WPAI work‐related items (OSA: absenteeism, *n* = 293; presenteeism, *n* = 293; work impairment, *n* = 288; narcolepsy: absenteeism, *n* = 140; presenteeism, *n* = 136; work impairment, *n* = 131).

**TABLE 1:** Demographic and baseline characteristics

Characteristic	Study	
	Obstructive sleep apnea^a^	Narcolepsy^b^
Sample size (*N*)	459	231
Gender (*n*, %)		
Female	172 (37.5%)	150 (64.9%)
Male	287 (62.5%)	81 (35.1%)
Age (years)		
Mean (SD)	53.86 (10.96)	36.20 (13.15)
Range	20–75	18–70
Region (*n*, %)		
Europe	15 (3.3%)	44 (19.0%)
North America	444 (96.7%)	187 (81.0%)
Race (*n*, %)		
American Indian	1 (0.2%)	2 (0.9%)
Asian	17 (3.7%)	6 (2.6%)
Black	87 (19.0%)	33 (14.3%)
Multiple	4 (0.9%)	5 (2.2%)
Pacific Islander	2 (0.4%)	1 (0.4%)
White	348 (75.8%)	184 (79.7%)
Ethnicity (*n*, %)		
Hispanic	40 (8.7%)	10 (4.3%)
Not Hispanic	419 (91.3%)	221 (95.7%)
Mean (SD) ESS score	15.2 (3.32)	17.2 (3.18)
Mean (SD) FOSQ−10 score	13.9 (3.01)	11.7 (3.03)

Note

ESS, Epworth Sleepiness Scale (possible scores range from 0 to 24, with higher scores reflecting more excessive daytime sleepiness); FOSQ‐10, Functional Outcomes of Sleep Questionnaire‐10 (possible scores range from 5 to 20, with higher scores representing better functioning); SD, standard deviation.

^a^ NCT02348606.

^b^ NCT02348593.

### 3.2 Correlations between measures of sleepiness, functioning and HRQoL at baseline

At baseline, correlations were low between the MWT or ESS and other functional and HRQoL measures with the exception of the correlation between the ESS and FOSQ‐10, for both participants with OSA (–0.476; *p* < 0.001) and participants with narcolepsy (–0.446; *p* < 0.001; Table 2).

**TABLE 2:** Pearson correlations between measures of sleepiness, functioning and health‐related quality of life at baseline

	MWT	ESS	FOSQ‐10	WPAI‐Abs	WPAI‐Pres	WPAI‐WPL	WPAI‐AI	SF‐36 MCS	SF‐36 PCS	SF‐36 Vitality	SF‐36 RP	EQ‐5D Utility Index
Participants with OSA												
MWT	1	−0.253	0.078	−0.050	−0.030	0.024	−0.002	0.014	0.074	0.028	0.050	0.097
Nominal *p*‐value^a^		<0.001	0.101	0.404	0.616	0.694	0.969	0.769	0.118	0.553	0.296	0.042
ESS	−0.253	1	−0.476	−0.003	0.164	0.177	0.185	−0.105	−0.112	−0.110	−0.135	−0.118
Nominal *p*‐value^a^	<0.001		<0.001	0.962	0.004	0.002	<0.001	0.023	0.014	0.017	0.003	0.010
Participants with narcolepsy												
MWT	1	−0.186	−0.082	0.176	−0.212	−0.191	−0.005	−0.028	−0.124	−0.144	−0.070	−0.057
Nominal *p*‐value^a^		0.005	0.221	0.041	0.016	0.033	0.936	0.674	0.064	0.031	0.297	0.401
ESS	−0.186	1	−0.446	0.269	0.246	0.287	0.235	−0.220	−0.208	−0.161	−0.271	−0.217
Nominal *p*‐value^a^	0.005		<0.001	0.001	0.004	0.001	<0.001	0.001	0.001	0.013	<0.001	0.001

Note

No shading, low correlation (absolute value, 0–0.3); light gray shading, moderate correlation (absolute value, 0.3–0.5).

Abbreviations: Abs, Absenteeism; AI, percent of activity impairment due to problem; EQ‐5D, EuroQoL 5‐Dimension; ESS, Epworth Sleepiness Scale; FOSQ‐10, Functional Outcomes of Sleep Questionnaire–short version; MCS, Mental Component Summary; MWT, Maintenance of Wakefulness Test; OSA, obstructive sleep apnea; PCS, Physical Component Summary; Pres, Presenteeism; RP, role physical; SF‐36, 36‐Item Short Form Health Survey; WPAI, Work Productivity and Activity Impairment questionnaire; WPL, percent of overall work impairment due to problem.

^a^ No adjustments for multiplicity were made; therefore, *p*‐values are nominal.

### 3.3 Correlations between changes in measures of sleepiness and changes in measures of functioning/HRQoL from baseline to week 12

**FIGURE 1:**
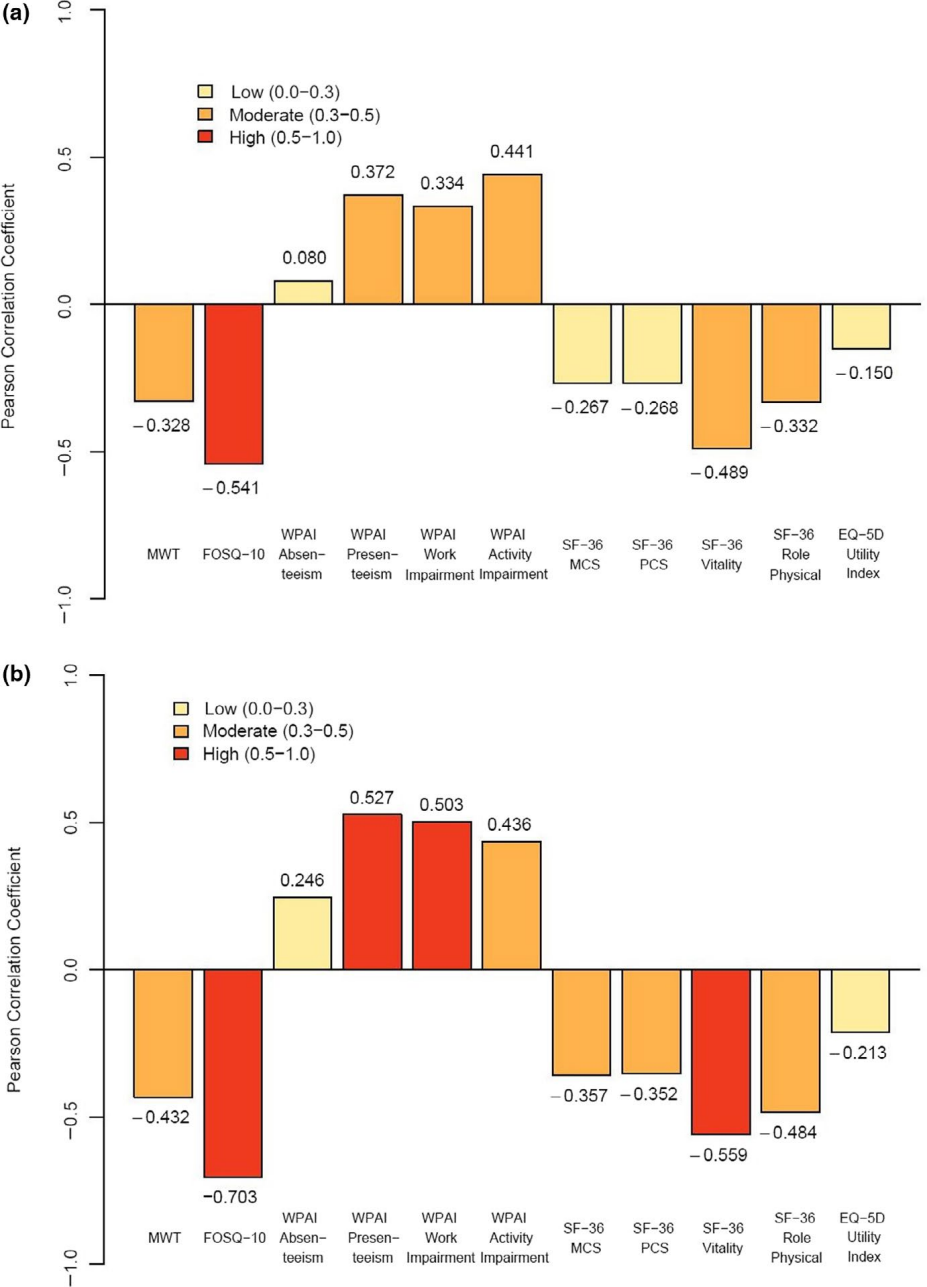
Correlations between change in ESS and MWT scores, measures of functioning, and health‐related quality of life from baseline to week 12 for participants with OSA (a) and narcolepsy (b). EQ‐5D, EuroQoL 5‐Dimension; ESS, Epworth Sleepiness Scale; FOSQ‐10, Functional Outcomes of Sleep Questionnaire–short version; MCS, Mental Component Summary; MWT, Maintenance of Wakefulness Test; OSA, obstructive sleep apnea; PCS, Physical Component Summary; SF‐36, 36‐Item Short Form Health Survey; WPAI, Work Productivity and Activity Impairment questionnaire

**FIGURE 2:**
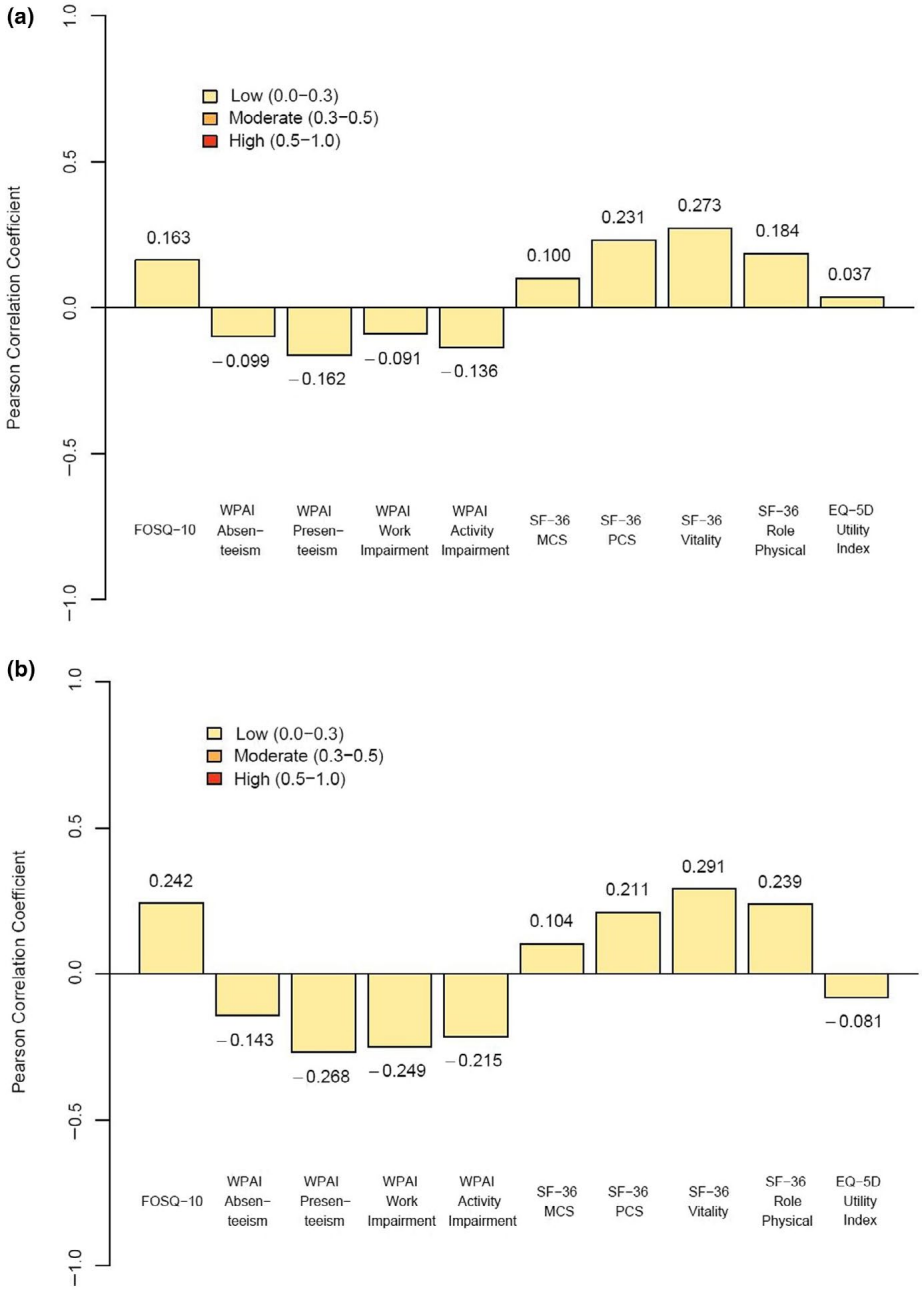
Correlations between change in MWT score and measures of functioning and health‐related quality of life from baseline to week 12 for participants with OSA (a) and narcolepsy (b). EQ‐5D, EuroQoL 5‐Dimension; FOSQ‐10, Functional Outcomes of Sleep Questionnaire–short version; MCS, Mental Component Summary; MWT, Maintenance of Wakefulness Test; OSA, obstructive sleep apnea; PCS, Physical Component Summary; SF‐36, 36‐Item Short Form Health Survey; WPAI, Work Productivity and Activity Impairment questionnaire

For participants with OSA, change in ESS score was highly correlated with change in the FOSQ‐10 (–0.541; *p* < 0.001) and moderately correlated with change in the MWT (–0.328; *p* < 0.001), SF‐36 Vitality (–0.489; *p* < 0.001) and role physical (–0.332; *p* < 0.001), and WPAI‐Presenteeism (0.372; *p* < 0.001), WPAI‐Work Impairment (0.334; *p* < 0.001) and WPAI‐Activity Impairment (0.441; *p* < 0.001). Correlations with changes in other measures were low (absolute value, 0.080–0.268; Figure 1a). Change in the MWT had low correlation (absolute value, 0.037–0.273) with change in all measures of functioning and HRQoL (Figure 2a).

For participants with narcolepsy, change in ESS score was highly correlated with change in the FOSQ‐10 (–0.703; *p* < 0.001), SF‐36 Vitality (–0.559; *p* < 0.001), and WPAI‐Presenteeism (–0.527; *p* < 0.001) and WPAI‐Work Impairment (0.503; *p* < 0.001) scores and moderately correlated with the MWT (–0.432; *p* < 0.001), SF‐36 Mental Component Summary (–0.357; *p* < 0.001), SF‐36 Physical Component Summary (–0.352; *p* < 0.001), SF‐36 role physical (–0.484; *p* < 0.001) and WPAI‐Activity Impairment (0.436; *p* < 0.001; Figure 1b) scores. Change in MWT scores had low correlations (absolute value, 0.081–0.291) with change in all measures of functioning and HRQoL (Figure 2b).

### 3.4 Correlations between measures of sleepiness, functioning and HRQoL at week 12

At week 12, correlations were moderate to high both in participants with OSA and in participants with narcolepsy between ESS and other functional HRQoL measures (absolute value, 0.302–0.665; *p* < 0.05) except for EQ‐5D Utility Index (–0.164, OSA; *p* = 0.001) and WPAI‐Absenteeism (OSA, 0.073, *p* = 0.225; narcolepsy, 0.247, *p* = 0.006), which were low; correlations between the MWT and other measures were low for both indications (absolute value, 0.004–0.276), except for two measures in participants with narcolepsy: WPAI‐Presenteeism (–0.349; *p*<0.001) and WPAI‐Work Impairment (–0.336; *p* < 0.001), for which they were moderate (see Table S1). The ESS and MWT were moderately correlated at week 12 for both indications (*p* < 0.001). PGI‐C ratings correlated highly with the ESS and FOSQ‐10 for both indications (absolute value, 0.504–0.584; *p* < 0.001) and with SF‐36 for participants with OSA (–0.581; *p* < 0.001). PGI‐C ratings correlated moderately with MWT, SF‐36 role physical, and WPAI‐Presenteeism, WPAI‐Work Impairment and WPAI‐Activity Impairment scores in both indications (absolute value, 0.331–0.477; *p* < 0.05) and with SF‐36 Mental Component Summary (–0.352; *p* < 0.001) and Physical Component Summary (–0.300; *p* < 0.001) scores in participants with OSA (Table S1).

## 4 DISCUSSION

The current study examines correlations between changes in measures of severity of EDS and those of functioning, productivity and HRQoL associated with OSA and narcolepsy. In both disorders, changes in severity on the ESS were most strongly correlated with FOSQ‐10 Total Score, SF‐36 Vitality, WPAI‐Presenteeism and WPAI‐Work Impairment. In general, correlations between change in the ESS and change in other measures were as high or higher in participants with narcolepsy compared with those with OSA. Other researchers have previously observed in participants with narcolepsy that treatment for EDS correlates most strongly with the vitality, role physical and social functioning domains of the SF‐36 (Bogan et al., 2016, 2017). Although we did not examine the correlation with the social functioning domain, our results are consistent with those found for the vitality and role physical domains, for which we observed moderate to high correlations between changes in ESS scores and changes in those domains. In the current study, changes in ESS scores had the weakest correlations with changes in EQ‐5D Utility Index scores and WPAI‐Absenteeism. Improvements in ESS scores were also moderately to highly correlated with improvements in WPAI scores, except for absenteeism (possibly due to its low base rate). Notably, almost 40% of study participants were not currently employed at baseline (39% of the narcolepsy study population, 36% of the OSA study population) and, therefore, were not included in the assessment of work‐related impairment. As a result, the assessment of absenteeism evaluated in the current analysis reflects work time missed among participants who were currently employed and does not take into account impairment among individuals who were unable to work due to their illness.

Correlations in baseline scores between either the ESS or MWT and all other measures were low, although previous studies have reported a moderate correlation between the ESS and FOSQ (Billings et al., 2014; Gooneratne et al., 2003). Despite these low correlations at baseline, moderate to high correlations were observed for *changes* in scores in the ESS and other measures. Correlations between the change in the MWT and changes in measures of functioning, work productivity and HRQoL were low for participants with OSA and those with narcolepsy, suggesting that although objective measures of sleepiness remain critical aspects of both assessment and treatment, subjective measures of the person’s experience are more reflective of trajectories of improvement in patient‐reported functioning.

The burden of EDS within these patient populations is considerable. EDS occurs in 100% of patients with narcolepsy and affects significant numbers of patients with OSA, including those currently receiving CPAP treatment (Koutsourelakis et al., 2009). Persistent EDS in patients with OSA treated with CPAP is associated with poorer emotional health and energy (Pepin et al., 2009), as well as depression (Koutsourelakis et al., 2009). Patients with narcolepsy who experience EDS report difficulties at work (completing detail‐oriented tasks, decreased productivity and making mistakes) and detriments to physical, emotional, cognitive and social health (Waldman et al., 2018a, 2018b), which are consistent with the current results demonstrated by patient responses to the WPAI, FOSQ‐10 and SF‐36. It is reasonable to hypothesize that reducing these EDS‐related issues is likely to have a significant impact on patients’ daily functioning. The participants in these two studies experienced significant improvements in the ESS, MWT and PGI‐C from baseline to week 12 at most doses (Schweitzer et al., 2019; Thorpy et al., 2019). The current results demonstrate that improvements in EDS with solriamfetol translated into improvements in work functioning and HRQoL. These results may help clinicians understand the relevance of levels of change in sleepiness and place them in the context of expected improvement in patient functioning, work productivity and HRQoL. Specifically, the FOSQ‐10 asks about aspects such as the ability to concentrate, operate a motor vehicle and participate in activities (with friends and work colleagues) during the morning and evening. Therefore, improvements in sleepiness are likely to reflect increased functioning in these areas, although they are distinct assessments.

This study fills a gap in the existing literature by examining the relationships between changes in sleep efficacy endpoints and functional status, work productivity and HRQoL as evaluated by several patient‐reported outcome measures in individuals with EDS associated with OSA and narcolepsy. However, there are limitations of our research that should be noted. First, all participants were randomly assigned to receive study treatment, and it is unclear to what extent current findings are generalizable to other patient populations with EDS. Second, there were no objective measures of functioning included (e.g., Psychomotor Vigilance Task, cognitive performance battery, or assessments of memory or driving performance). Such objective measures may correlate more highly with objective assessments of ability to stay awake, such as the MWT (Pizza et al., 2009). Future studies should explore the relationship of these objective measures. Additionally, no depression‐specific measures were assessed; however, a dimension of anxiety/depression is included in the EQ‐5D, and mental health and social functioning domains are included in the SF‐36 Mental Component Summary, thus providing insight into the relationship between changes in EDS and mood symptoms. Further, there are similarities between the types of questions included in the ESS and other patient‐reported outcome questionnaires. For instance, both the ESS and the FOSQ ask questions related to driving; however, the formats of the questions differ in ways that reflect differences in the conceptual bases for the development of the instruments. For example, the ESS asks about the likelihood of falling asleep while driving, whereas the FOSQ asks about the level of driving difficulty attributable to sleepiness. Therefore, although both questions relate to driving, the ESS question is a measure of the severity of sleepiness, whereas the FOSQ question is a measure of the impairment associated with the sleepiness. A final limitation is that correlation coefficients were not adjusted for multiple comparisons.

In adults with OSA or narcolepsy treated for EDS, we observed that changes in measures of sleepiness (ESS and MWT) were correlated with measures of functioning and HRQoL status (FOSQ‐10, SF‐36, EQ‐5D and WPAI) to varying degrees. Further, the magnitudes of the correlations between the change in the self‐reported sleepiness (ESS) and changes in measures of functioning and HRQoL were stronger than those between the objective measure of sleepiness (MWT) and these measures. Week 12 correlations with these measures were also stronger for the ESS and PGI‐C than for the MWT. Taken together, these results suggest that self‐reported assessments of sleepiness may be more strongly associated with functional and HRQoL outcomes than objectively assessed sleepiness.

## CONFLICT OF INTEREST

TEW receives a royalty fee for use of the FOSQ from Philips Respironics, Nyxoah, Bayer AG, ResMed, ResMed Germany, Jazz Pharmaceuticals, Cook Medical, RWS, Verily Life Sciences, Stratevi, WCG MedAvante Prophase and Merck Co, Inc; SDM is an employee of Health Outcomes Solutions (HOS), which received funding from Jazz Pharmaceuticals for analyzing these data; RDC is a consultant to HOS, which received funding from Jazz Pharmaceuticals for analyzing these data; SB is an employee of Jazz Pharmaceuticals and holds stock and/or stock options in Jazz Pharmaceuticals, PLC; MB, DM and KFV were employees of Jazz Pharmaceuticals at the time this study was conducted and hold stock and/or stock options in Jazz Pharmaceuticals, PLC; CD is a consultant to Jazz Pharmaceuticals and has received research support from Jazz Pharmaceuticals.

## AUTHOR CONTRIBUTIONS

All authors collaborated in the preparation of the manuscript and critically reviewed and provided revisions to the paper. All authors had access to the data and assume responsibility for the completeness and accuracy of the data and data analyses. All authors gave final approval of the manuscript for submission.

## Supporting information

Table S1

## Data Availability

All relevant data are provided within the manuscript and supporting files.
