# Supplementary material for: Relationship between sleep efficacy endpoints and measures of functional status and health‐related quality of life in participants with narcolepsy or obstructive sleep apnea treated for excessive daytime sleepiness
Source: J Sleep Res. 2020 Oct 13;30(3):e13210. doi: 10.1111/jsr.13210 (PMC8244115; doi:10.1111/jsr.13210)
Supplement: Supplementary file 1 — Table S1 [file JSR-30-e13210-s001.docx]

**Supporting Information**

Supplemental Table S1. Pearson correlations between measures of sleepiness, functioning, and health-related quality of life at Week 12

|  | MWT | ESS | FOSQ‑ 10 | WPAI- Abs | WPAI- Pres | WPAI- WPL | WPAI- AI | SF-36 MCS | SF-36 PCS | SF-36 Vitality | SF-36 RP | EQ-5D Utility Index |
| --- | --- | --- | --- | --- | --- | --- | --- | --- | --- | --- | --- | --- |
| Participants with OSA | | | | | | | | | | | | |
| MWT | 1 | −0.418 | 0.276 | −0.114 | −0.251 | −0.258 | −0.251 | 0.013 | 0.222 | 0.221 | 0.211 | 0.055 |
| Nominal *P*-value^a^ |  | <0.001 | <0.001 | 0.063 | <0.001 | <0.001 | <0.001 | 0.790 | <0.001 | <0.001 | <0.001 | 0.271 |
| ESS | −0.418 | 1 | −0.633 | 0.073 | 0.446 | 0.359 | 0.482 | −0.306 | −0.302 | −0.506 | −0.382 | −0.164 |
| Nominal *P*-value^a^ | <0.001 |  | <0.001 | 0.225 | <0.001 | <0.001 | <0.001 | <0.001 | <0.001 | <0.001 | <0.001 | 0.001 |
| PGI-C | −0.339 | 0.584 | −0.529 | 0.180 | 0.449 | 0.398 | 0.476 | −0.352 | −0.300 | −0.581 | −0.371 | −0.188 |
| Nominal *P*-value^a^ | <0.001 | <0.001 | <0.001 | 0.003 | <0.001 | <0.001 | <0.001 | <0.001 | <0.001 | <0.001 | <0.001 | <0.001 |
| Participants with narcolepsy | | | | | | | | | | | | |
| MWT | 1 | −0.496 | 0.183 | −0.127 | −0.349 | −0.336 | −0.212 | 0.083 | 0.053 | 0.162 | 0.177 | 0.004 |
| Nominal *P*-value^a^ |  | <0.001 | 0.011 | 0.180 | <0.001 | <0.001 | 0.003 | 0.254 | 0.467 | 0.026 | 0.015 | 0.960 |
| ESS | −0.496 | 1 | −0.665 | 0.247 | 0.565 | 0.476 | 0.557 | −0.347 | −0.378 | −0.486 | −0.520 | −0.316 |
| Nominal *P*-value^a^ | <0.001 |  | <0.001 | 0.006 | <0.001 | <0.001 | <0.001 | <0.001 | <0.001 | <0.001 | <0.001 | <0.001 |
| PGI-C | −0.331 | 0.561 | −0.504 | 0.215 | 0.453 | 0.417 | 0.477 | −0.268 | −0.297 | −0.499 | −0.405 | −0.243 |
| Nominal *P*-value^a^ | 0.000 | 0.000 | 0.000 | 0.020 | 0.000 | 0.000 | 0.000 | 0.000 | 0.000 | 0.000 | 0.000 | 0.001 |

No shading, low correlation (absolute value, 0-0.3); light grey, moderate correlation (absolute value, 0.3-0.5); dark grey, high correlation (absolute value, >0.5).

^a^No adjustments for multiplicity were made; therefore, *P*-values are nominal.

Abs, Absenteeism; AI, percent of activity impairment due to problem; EQ-5D, EuroQoL 5-Dimension; ESS, Epworth Sleepiness Scale; FOSQ-10, Functional Outcomes of Sleep Questionnaire–short version; MCS, Mental Component Summary; MWT, Maintenance of Wakefulness Test; OSA, obstructive sleep apnea; PCS, Physical Component Summary; PGI-C, Patient Global Impression of Change; Pres, Presenteeism; RP, role physical; SF-36, 36-Item Short Form Health Survey; WPAI, Work Productivity and Activity Impairment Questionnaire; WPL, percent of overall work impairment due to problem.
